# Reply to Bratchenko and Bratchenko, “Overestimation of the classification model for Raman spectroscopy data of biological samples”

**DOI:** 10.1128/msystems.00103-25

**Published:** 2025-03-10

**Authors:** Xin-Ru Wen, Jia-Wei Tang, Liang Wang

**Affiliations:** 1 School of Medical Informatics and Engineering Xuzhou Medical University, Xuzhou, Jiangsu, China; 2 Department of Laboratory Medicine, Guangdong Provincial People’s Hospital (Guangdong Academy of Medical Sciences), Southern Medical University, Guangzhou, China; 3 Division of Microbiology and Immunology School of Biomedical Sciences, The University of Western Australia, Crawley, Western Australia, Australia; 4 Centre for Precision Health, School of Medical and Health Sciences, Edith Cowan University, Joondalup, Western Australia, Australia; Katholieke Universiteit Leuven, Leuven, Belgium

## REPLY

We appreciate the interest of Prof. Ivan A. Bratchenko and Associate Professor Lyudmila A. Bratchenko in our work and sincerely thank them for commenting on our recent study about the rapid diagnosis of bacterial vaginosis (BV) using machine-learning-assisted surface-enhanced Raman spectroscopy of human vaginal fluids (1).

In the comment, it was pointed out that “despite the high accuracy of the suggested approach (99%), the demonstrated accuracy could be treated as overoptimistic due to classification models overestimation.” In addition, the commentators also inferred that “one possible reason for the model’s overestimation is the collection of multiple spectra from one sample,” through which “the classification model is already familiar with the validation data and will demonstrate an excellent result.” Finally, the commentators suggested that “only the value of accuracy obtained for the unknown test data may be utilized as the true accuracy value for the proposed classifier.”

**Fig 1:**
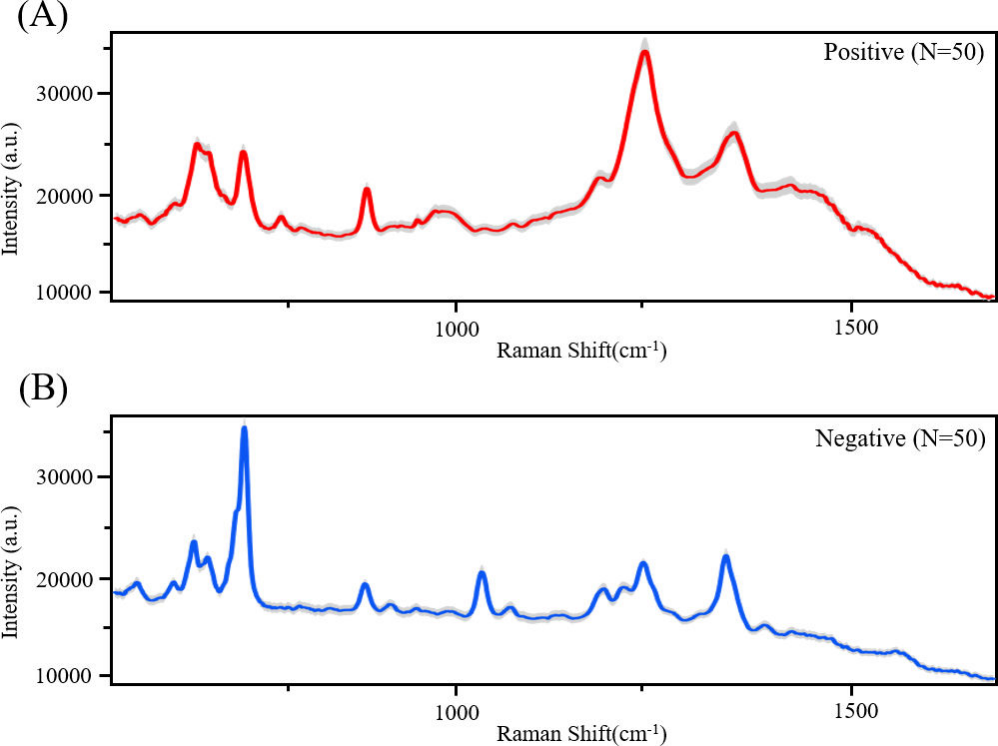
Representative average SERS spectrum of (**A**) a BV-positive sample and (**B**) a BV-negative sample, respectively. Each average spectrum was generated via 50 SERS spectra from a single sample. The gray region represents 20% of the standard deviation of the repeated spectra at each Raman shift. *X*-axis: Raman shift (cm^−1^). *Y*-axis: Raman signal intensities with arbitrary units.

First of all, we would like to clarify the procedure of the surface-enhanced Raman spectroscopy (SERS) spectral collection. In this study, the inVia Raman microscope (Renishaw Plc., New Mills, Wotton-under-Edge, UK) was used to collect Raman signals. Each vaginal fluid sample was dried on a silicon wafer to form a dried spot, and for each sample spot, 50 different loci were randomly selected for SERS signal detection through automated scanning of the spectrometer. In this manner, we did collect multiple spectra from one sample. However, these SERS spectra are better at representing a vaginal fluid sample due to the heterogeneity of a dried spot. Below is a representative average SERS spectrum of 50 SERS spectra generated from BV-positive and BV-negative vaginal fluid samples, respectively (Fig. 1). The gray region represents a 20% standard deviation of these spectra at each Raman shift, indicating variations among spectra from the same sample. This suggests that collecting multiple SERS spectra from one sample is reasonable. The SERS technique has been applied to many clinical samples for research purposes of disease diagnosis (2). It is not rare to collect repeated spectra from the same sample by other published studies, mainly because of the extreme sensitivity of surface-enhanced Raman spectroscopy, leading to the variations of SERS spectra from the same sample. For example, Ho et al. collected 100 spectral data points for each clinically common bacterial strain to construct a reference database (3). Lee et al. measured 20 spectral data points for each patient’s serum sample to evaluate the analytical capability of SERS in distinguishing rejection types in kidney transplant patients and identifying molecular element variations (4). Huang et al. combined Raman spectroscopy with deep learning to differentiate liver cancer tissues from adjacent lung tumor tissues, collecting at least 50 spectral data points for each patient’s tissue sample (5).

Second, we agree with the commentators that the high accuracy of the trained model at 99% can be overestimated due to the possibility of utilizing SERS spectral data from one sample both in training and validation. During the model training process, we employed 5-fold cross-validation to minimize the risk of overfitting. However, since spectral data from the same sample could be simultaneously split into both the training set and the validation set, the model still exhibited overestimation. Considering the overoptimistic potential, we applied the trained model to a group of independent samples (*N* = 40) with unknown bacterial vaginosis status for a blind test. Results in the original manuscript showed that the optimized convolutional neural network (CNN) model achieved an average prediction accuracy for BV-positive vaginal fluid samples at 90.75% and for BV-negative vaginal fluid samples at 87.25%. Therefore, while we acknowledge the concerns raised by the reviewers, we believe that an independent external validation data set can effectively address this issue. In the abstract, we also emphasized the blind test result in the sentence, “CNN model was blindly tested on SERS spectra of vaginal fluid samples collected from 40 participants with unknown BV infection status, achieving a prediction accuracy of 90.75% compared with the results of the BVBlue Test combined with clinical microscopy.” Here, in this response letter, as suggested by Prof. Ivan A. Bratchenko and Associate Professor Lyudmila A. Bratchenko, we would also like to emphasize that the blind test accuracy should be considered the true accuracy value for the proposed CNN classifier.

Overall, we thank Prof. Ivan A. Bratchenko and Associate Professor Lyudmila A. Bratchenko for their constructive comments on this study, which clarified the predictive accuracy of the model. For constructing a more accurate and robust model for potential clinical application, the future work will be collecting more high-quality SERS spectra of vaginal fluid samples via a multicenter, prospective, and randomized study, through which a predictive model with better-performing capacities in real-world settings, for example, hospitals, clinical laboratory, etc, shall be achieved.
